# Effects of rs7903146 Variation in the *Tcf7l2* Gene in the Lipid Metabolism of Three Different Populations

**DOI:** 10.1371/journal.pone.0043390

**Published:** 2012-08-20

**Authors:** Pablo Perez-Martinez, Ana I. Perez-Caballero, Antonio Garcia-Rios, Elena M. Yubero-Serrano, Antonio Camargo, Maria J. Gomez-Luna, Carmen Marin, Purificacion Gomez-Luna, Aldona Dembinska-Kiec, Fernando Rodriguez-Cantalejo, Francisco J. Tinahones, Helen M. Roche, Francisco Perez-Jimenez, Jose Lopez-Miranda, Javier Delgado-Lista

**Affiliations:** 1 Lipids and Atherosclerosis Unit, IMIBIC/Hospital Universitario Reina Sofía/Universidad de Cordoba, Cordoba, Spain; 2 CIBER Fisiopatologia Obesidad y Nutricion (CIBEROBN), Instituto de Salud Carlos III, Madrid, Spain; 3 Department of Clinical Biochemistry, Jagiellonian University Medical College and the Off-Patients Clinic for Dyslipidemia and Obesity Treatment, Krakow, Poland; 4 Biochemical Laboratory, Hospital Universitario Reina Sofía, Cordoba, Spain; 5 Servicio de Endocrinología y Nutrición, Hospital Clínico Virgen de la Victoria, Málaga, Spain; 6 Nutrigenomics Research Group, UCD School of Public Health and Population Science, UCD Conway Institute, University College Dublin, Dublin, Ireland; University of Oxford, United Kingdom

## Abstract

**Background:**

*TCF7L2* rs7903146 is an important genetic factor predicting type 2 diabetes (T2DM) which has also been linked to higher cardiovascular risk. To date, there is little information about the additional impact of this single nucleotide polymorphism (SNP) beyond glucose metabolism.

**Methodology/Principal Findings:**

We studied whether rs7903146 influenced postprandial lipid metabolism in three different populations (healthy young men, metabolic syndrome (MetS) patients and elderly persons). Eighty-eight healthy males were submitted to a single saturated fatty acid-rich test meal. Additionally, 110 middle-aged MetS patients and 20 healthy elderly persons (≥65 years) were submitted to three different dietary models followed by test meals. Minor allele homozygotes for rs7903146 showed a worse postprandial lipemia profile in young males, as seen by a lower HDL-cholesterol and Apo A1 concentration during the postprandial lipemia and a trend towards higher triglycerides (TG), than the other genotypes. In healthy elderly persons, carriers of the minor allele showed higher total cholesterol, LDL-cholesterol, Apo B and TG in the fasting state, and a higher postprandial area under the curve for total cholesterol, Apo B, small-triglyceride rich lipoprotein (TRL) cholesterol and small-(TRL) triglycerides. These results were accompanied by differential changes in adipokines. We did not observe any influence of rs7903146 on the postprandium of MetS patients.

**Conclusions/Significance:**

Healthy young males and elderly persons who are carriers of the mutant allele for rs7903146 have an impaired postprandial lipid metabolism that may be mediated by an alteration in adipokine regulation, and may be related to the higher cardiovascular risk observed in these persons.

**Trial Registration:**

ClinicalTrials.gov NCT00429195

## Introduction

Postprandial lipemia is characterized as a period by an increase in proinflammatory and prooxidant species in the bloodstream, along with a transient increase in some lipid fractions with direct pro-atherogenic properties, such as small triglyceride-rich lipoproteins (TRL). Several studies have linked the extent of this state to the development of coronary heart disease [1]–[3]. How long the state lasts varies widely among healthy individuals, and both intrinsic and extrinsic factors have been identified, with genetic background and diet those factors which have been most extensively studied [4]–[10]. Several diseases have also been recognized as powerful modifiers of postprandial lipemia, such as type 2 diabetes (T2DM), and metabolic syndrome (MetS). These two conditions present an altered lipid metabolism, with increased triglyceride levels and increased postprandial lipemia, along with lower HDL levels and a proatherogenic LDL phenotype consisting of smaller and denser molecules. The combination of all these features makes up what is called atherogenic (or diabetic) dyslipidemia [11]–[12].

Variations at the *TCF7L2* (a subgroup of T-cell-specific transcription factors) locus have been identified as the most important genetic predictors of T2DM in Genome-wide association studies [13]–[15]. TCF7L2 are transcriptor factors, main effectors of the Wnt ligand family, which have been involved in the differentiation of adipocytes, the regulation of adipokines and pancreatic beta-cell function, among others [16].

We recently reported how a variation of the rs7903146 gene is linked to a dysfunction in glucose metabolism during the postprandial state in MetS patients, without affecting fasting glucose or insulin, and mainly influencing markers of beta-cell function, such as those associated to insulin secretion [17].

In contrast to the currently burgeoning data on the influence of *TCF7L2* variations on glucose metabolism, which is the subject of over 80 studies, their influence on postprandial lipids is at present unexplored, with only one study reporting an altered postprandial lipid profile in consumers of high polyunsaturated fatty acids (PUFA) [18]. Furthermore, neither the effects on different populations nor their possible underlying mechanisms (beyond the beta-cell function) have been explored, despite the well-known regulation of adipocyte differentiation by *TCF7L2*.

In this study, we aimed to explore the influence of rs7903146 on postprandial lipid metabolism in different populations and under different dietary models, and relate our findings to possible underlying mechanisms, by assessing both beta-cell function and the regulation of adipokines.

With this aim, we first conducted a closely-controlled intervention study of a group of young men, to assess the effect of the SNPs on postprandial lipemia in a healthy cohort, where modification due to beta-cell dysfunction was unlikely, and which has been identified as a good model to test for new gene variations affecting postprandial lipemia [6]–[10], [19]. Then, to corroborate our findings, we explored the effects of the SNP on two other populations: one with MetS and another made up of healthy elderly persons.

## Methods

### Ethics

In the three studies (young men, MetS and healthy elderly persons), all participants signed an informed consent agreement. The experimental protocols were approved by the local ethics committees at the Bioethical Committee of the Jagiellonian University, Medical College Krakow, Poland and the Comité Ético de Investigación Clínica, Hospital Universitario Reina Sofía, Cordoba, Spain, according to the Helsinki Declaration of 1975 (revised in 1983).

### Young Male Population and Fat-loading Test

Eighty-eight healthy male students at the University of Cordoba were recruited for the study. They had a mean ±SD age of 23±4 y. Other studies reporting exhaustive baseline characteristics of this population have been published elsewhere [7], [9]–[10], [19]–[20]. Briefly described, participants were free of any known condition when recruited for the study, and were not taking any medication. They had plasma total cholesterol concentrations <240 mg/dL, plasma triacylglycerol concentrations <150 mg/dL, and BMI<30 kg/m^2^. The metabolic study was carried out in the Research Unit of the Reina Sofia University Hospital. After a 12-h fast, volunteers were given a fatty meal. The amount of fat given, per kg body wt, was 1 g fat and 7 mg cholesterol. The meal contained 65% of energy as fat, 10% of energy as protein, and 25% of energy as carbohydrates; it was consumed in 20 min. After this meal, subjects fasted for 11 h, but they were allowed to drink water. Blood samples were drawn before the meal, every hour until hour 6, and every 2.5 h until hour 11. Extensive methodology has been published [7].

### MetS Population and Fat-loading Test

This study was carried out within the LIPGENE study. A total of 117 patients with MetS from the Lipid and Atherosclerosis Unit at the IMIBIC/Reina Sofia University Hospital, Spain, and the Department of Clinical Biochemistry, Jagiellonian University School of Medicine, Poland, participated in this sub-study [21]–[22].

Patients were randomly stratified to 1 of 4 dietary interventions for 12 wk, as published [21]–[22]. In brief, there were four isoenergetic diets which differed in fat quantity and quality. Two diets were designed to provide 38% energy (E) from fat: a high-fat, saturated fatty acids-rich diet (HSFA), which was designed to provide ∼16% E as SFA, and a a high-fat, monounsaturated fatty acids-rich diet (HMUFA) diet designed to provide ∼20% E from MUFA. The other 2 diets were low fat, high carbohydrates (LFHCC) diets (LFHCC and LFHCC (n-3); 28% E from fat); the LFHCC (n-3) diet included a 1.24-g/d supplement of long chain (n-3) PUFA [ratio of 1.4 eicosapentaenoic acid (EPA):1 docosahexaenoic acid (DHA)] in form of capsules and the LFHCC diet included a 1.24-g/d supplement of control high-oleic sunflower seed oil capsules (placebo). The fat-loading test was performed pre- and post-intervention, providing the same amount of fat (0.7 g/kg body weight), the fat composition of which reflected the amount consumed within the intervention period. Blood samples were taken at 0,2,4,6 and 8 hours after intake. Test meals provided an equal amount of fat (0.7 g/kg body weight), energy content (40.2 kJ/kg body weight) and cholesterol (5 mg/kg of body weight). The test meal provided 65% of E as fat, 10% as protein, and 25% as carbohydrates. During the postprandial assessment, the participants rested and did not consume any other food for 9 h, but were allowed to drink water. The composition of the test meals was as follows: HSFA, 38% E from SFA; HMUFA, 43% E from MUFA; LFHCC with placebo capsules, 16% E as PUFA; LFHCC with long chain (n-3) PUFA, 16% E as PUFA [1.24 g/d of long chain (n-3) PUFA (ratio 1.4 EPA:1 DHA)].

### Elderly Population and Fat-loading Test

Twenty elderly (≥65 years) persons, 10 men and 10 women, entered the study. They had total cholesterol concentration equal to or <250 mg/dl, and were non-smokers. The clinical exclusion criteria were age <65 years, diabetes or other endocrine disorders, chronic inflammatory conditions, kidney or liver dysfunction, iron deficiency anemia (hemoglobin <12 g/dL men, <11 g/dL women), prescribed hypolipidemic and anti-inflammatory medication, fatty acid supplements including fish oil, consumers of high doses of antioxidant vitamins (A, C, E, β-carotene), highly-trained or endurance athletes or those who participated in more than three periods of intense exercise per week, weight change equal or >3 kg within the last 3 months, smokers, and subjects with alcohol or drug abuse (based on clinical judgment).

The participants were randomly assigned to receive, in a crossover design, three isocaloric diets for a 4-week period each. The three diets were as follows: (1) A Mediterranean diet supplemented with coenzyme Q (Med+CoQ diet) (200 mg/day in capsules), containing 15% of energy as protein, 47% of energy as carbohydrate and 38% of total energy as fat [24% MUFA, provided by virgin olive oil, 10% SFA, 4% PUFA]. (2) A Mediterranean diet not supplemented with CoQ (Med diet), with the same composition of the first diet, but supplemented by placebo capsules, and (3) A Western diet rich in SFA, with 15% of energy as protein, 47% of energy as carbohydrate, and 38% of total energy as fat (12% MUFA, 22% SFA, 4% PUFA). The amount of fat given, per kg body wt, was 0.7 g fat and 5 mg cholesterol. At the end of the dietary intervention period, the subjects were given a fatty test meal with the same fat composition as consumed in each of the diets. Subsequent blood samples were taken at 0, 2 and 4 h. The meals contained 65% of energy as fat, 10% of energy as protein, and 25% of energy as carbohydrates, were consumed in 20 min, and their fatty acid composition was as follows: Med with CoQ (400 mg in capsules) test meal (12% SFA, 43% MUFA, 10% PUFA); Med with placebo capsule test meal (12% SFA, 43% MUFA, 10% PUFA); and SFA-rich test meal (38% SFA, 21% MUFA, 6% PUFA).

### Laboratory Methods

Laboratory methodology has been published extensively for the three populations elsewhere [7], [23]–[24]. Beta-cell function was explored by the HOMA-B method (360*fasting insulin in mU/mL)/(fasting glucose in mg/dL-63)% [25]. Blood collection and TRL (Large and Small) isolation were performed by standard methodology, as previously published [7] (**File S1**). Total cholesterol (Chol) and triglycerides (TG) in plasma and lipoprotein fractions were assayed by enzymatic procedures. Apo A1 and Apo B were determined by turbidimetry [26]. HDL-C was measured by the dextran sulfate-Mg^2+^ method, as described [27]. LDL-C levels were estimated using the Friedewald formula [28] for the healthy young men and the MetS cohorts, and calculating the difference between the total cholesterol before ultracentrifugation and that at the bottom part of the tube after ultracentrifugation at a density of 1019 kg/L for the elderly persons cohort. Plasma concentrations of adiponectin, leptin and resistin were measured by enzyme-linked immunosorbent assay by the Milliplex MAP kit (Millipore Corporation, Billerica, MA, USA) using a Bio-Plex 200 System (BIORAD Laboratories Hercules, CA, USA).

### Methodology for Choosing and Genotyping the *TCF7L2* Gene Variant

We chose rs7903146 as a good candidate for studying the phenotypical effects on lipids of *TCF7L2* based on strong indications in previous literature (more than 175 articles reporting phenotypical associations), some of which suggested that its functionality affected glucose metabolism [29]–[32]. Furthermore, the only article reporting the postprandial triglyceride effects of *TCF7L2* involved rs7903146 [18]. Genotyping of the young male cohort and the elderly persons cohort was performed by Progenika inc. (Medford, MA 02155 USA). Genotyping of the MetS population was performed by Illumina Inc., (San Diego, CA, USA). The Hardy-Weinberg equilibrium (HWE) was tested by Fisheŕs exact test.

### Statistical Analysis

The statistical analysis methods employed here are similar to those which we have published previously with respect to the gene-postprandial state interaction [9]–[10], [19].

Analysis of lipid parameters and genotype.

The influence of the SNP on the size of the postprandial lipid fractions was analyzed by univariate ANOVA for area under the curve (AUC), defined as the area between the plasma concentration-versus-time curve, using the trapezoidal rule, with the SNP included as an independent factor, and BMI, center of origin, gender, age and diet (if applicable) as covariates. When we found significant data in the univariate ANOVA, we performed repeated-measures ANOVA for both the overall gene influence (global ANOVA, p for gene influence), the kinetics of the response (p for time), and the interaction of both factors (time*gene). We used the dominant genetic model as the default model, based on previous data [17]. We also performed the additive model in the young cohort, inferring that in such a population a higher genetic challenge might be necessary to produce phenotypic findings.

A p-value of less than 0.05 was considered significant. All data presented in the text and tables are expressed as mean ±S.E, unless otherwise specified. SPSS 19.0 for Windows (SPSS Inc., Chicago, IL, USA) was used for statistical comparisons.

## Results

The baseline of the participants of the three cohorts is reported in **Table 1**. In the cohort of elderly persons, total cholesterol, TG, LDL and Apo B were higher in the fasting state in the carriers of the rs7903146 than in the homozygotes for the major allele. We did not find any other differences in baseline characteristics between CC and CT/TT in either of the other two cohorts. The minor allele frequencies in the young men, MetS and elderly cohorts were 0.39, 0.31, and 0.32. All the frequencies were in the Hardy-Weinberg equilibrium. As an internal control, we used Bootstraping methods to confirm the main findings of this article. Bootstrapping test used the following specifications: Simple sampling method, 10,000 permutations per test and 95% of confidence interval. C.I.95%: Difference of means. In all cases, Boostrapping confirmed the results from ANOVA test. **File S2** shows this information.

**Table 1:** Baseline characteristics of the participants in the three studies depending on the TCF7L2 rs7903146 SNP.

	Young men				Elderly persons			Metabolic Syndrome		
	CC (n = 34)	CT (n = 39)	TT (n = 15)	*p*	CC (n = 10)	CT/TT (n = 10)	*p*	CC (n = 55)	CT/TT (n = 55)	*p*
Age (years)	22.7±0.7	22±0.3	21.6±0.7	0.378	67.5±1.6	68.4±1.7	0.419	54.2±0.9	55.6±0.9	0.261
BMI (kg/m^2^)	25.3±0.6	25.9±0.7	24.8±1	0.594	33.1±2.2	30.1±1.4	0.26	34.5±0.4	34.7±0.4	0.75
Weight (kg)	78.1±1.9	78.4±2.1	74.3±2.3	0.463	88.8±5.7	77.6±4.8	0.15	93.9±1.6	91.5±1.4	0.264
Height (cm)	175.7±0.9	173.9±1.2	173.3±1.4	0.329	163.9±2.6	159.4±2.6	0.244	170.1±0.9	1.64±3.0	0.128
Chol (mg/dl)	154.6±3	156.5±4.5	145.7±5.8	0.059	**185.8**±**8.7**	**216.8**±**7.1**	**0.013** **	211±5.2	207.6±4.7	0.458
TG (mg/dl)	82.2±7.3	82.3±5.8	72.8±6.1	0.635	**87.2**±**11.6**	**117.9**±**7.7**	**0.043** **	151.6±7.7	151.4±10.9	0.984
HDL (mg/dl)	47.6±1.9	47.5±1.8	43±1.4	0.073	55.7±3.5	52.3±4.4	0.56	44.7±1.5	44±1.4	0.725
LDL (mg/dl)	90.6±3.1	92.5±4.4	81.2±4.9	0.228	**109**±**7.8**	**137.5**±**8.6**	**0.024** **	142.9±4.5	139.2±4.1	0.452
Apo A1 (mg/dl)	106±3.8	111±3.2	99.8±2.6	0.056	157.3±9.1	154±8.4	0.795	140.3±2.9	138.3±3.2	0.653
Apo B (mg/dl)	67.6±2.9	68.1±3	66.3±4	0.934	**78.8**±**5.8**	**102.6**±**5.8**	**0.009** **	99.1±2.7	97.1±2.6	0.463

Chol: total cholesterol. All values are means ±S.E.

* p<0,05 TT vs CT/CC;

** p<0.05 CC vs CT/TT. Adjusted by age and BMI (all cohorts), diet and gender (Metabolic syndrome and aged cohorts), and center of origin (Metabolic Syndrome).

### Young Male Population and Fat-loading Test

**Table 2** shows the area under the curve (AUC) of the different lipid fractions after the test meal. Homozygotes for the rare allele (TT) of rs7903146 exhibited a lower AUC than the other two genotypes for HDL (p = 0.028), and Apo A1 (p = 0.011). There was also a trend towards lower total cholesterol AUC in TT, although it did not reach statistical significance (p = 0.053).

**Table 2:** AUC of the different lipid particles in the young male cohort (min*mg/dL)/10^3^.

Young male cohort	Rs7903146			*P*
Lipemia (11 hours)	CC (n = 34)	CT (n = 39)	TT (n = 15)	
Total TG	89.0±7.1	90.2±6.9	86.9±10.6	0.967
CHOL	98.5±2.5	102.5±2.4	91.5±3.7	0.053
large-TRL TG	34.8±3.5	35.0±3.3	36.5±5.3	0.966
small-TRL TG	30.6±1.7	31.8±2.3	29.7±1.4	0.964
large-TRL CHOL	5.3±0.3	5.6±0.3	5.2±0.5	0.786
small-TRL CHOL	7.3±0.7	7.6±0.6	7.1±1.0	0.915
APOA1	**67.9±1.8**	**70.8±1.8**	**60.9±2.7** **	**0.011**
Apo B	41.5±1.6	44.2±1.6	44.3±2.4	0.452
HDL	**30.54±1.1**	**30.0±1.6**	**25.3±1.6** *	**0.028**

* p<0.05 TT vs CT and TT vs CC.

** p<0.05 TT vs CT. All values are mean±S.E. Adjusted by age and BMI.

When evaluating the timepoint effects (repeated measures), we found differences for HDL at all measurements except at the 6^th^ hour (timepoints: 0, 1, 2, 3, 4, 5, 8.5 and 11 hours). Significance for differences at 6^th^ hour were p = 0.080 and p = 0.086 for TT vs CC and CT respectively. For Apo A1, the results were similar, with differences at all timepoints between TT and CT and at all except timepoint 6 hours for TT vs CC (p = 0.142).

**Figure 1:**
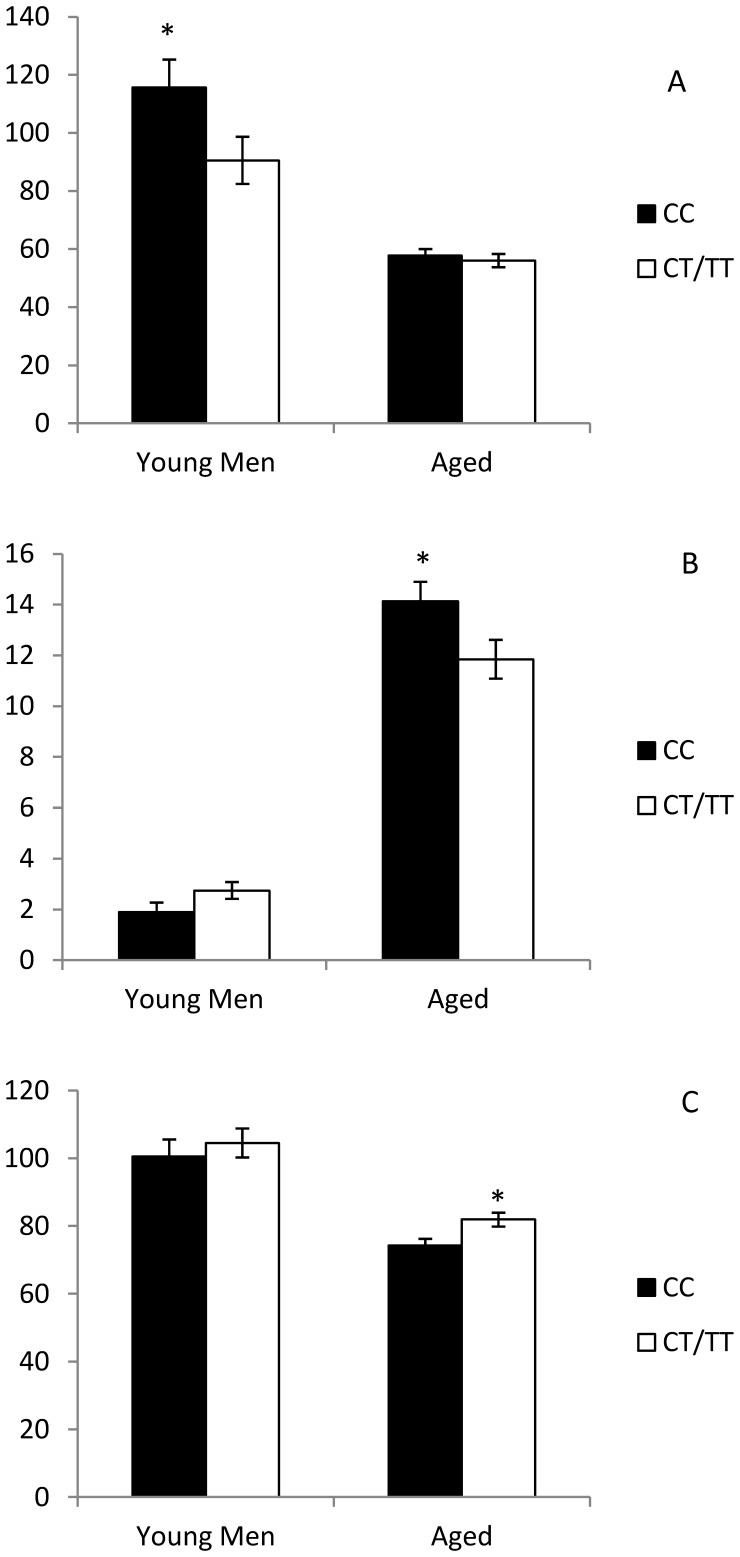
Plasma concentration of adiponectin (Panel A), leptin (Panel B) and resistin (Panel C) depending on the rs7903146 genotype. *p<0.05 CC vs CT/TT.

AUC of Adiponectin was higher, and leptin lower, in the homozygotes for the major allele (all p<0.05), **Figure 1**. No differences were found for HOMA-B index (p>0.05, data not shown).

### MetS Population and Fat-loading Test

We did not find any significant differences in the different lipid particles analyzed, depending on the rs7903146 genotype. There were non-significant higher means for TG, large-TRL TG, and lower means for Apo A1 in the carriers of the mutant allele (**Table 3**). HOMA-B index was analyzed previously in a sample that contained this cohort in a previous report, and a lower HOMA-B index was observed in the carriers of the minor alleles [17].

**Table 3:** AUC of the different lipid particles in the metabolic syndrome cohort under the dominant model assumption (min*mg/dL)/10^3^.

Metabolic syndrome cohort	Rs7903146		*P*
Lipemia (8 hours)	CC (n = 55)	CT/TT (n = 55)	
Total TG	97.2±7.3	104.4±6.8	0.483
CHOL	94.3±2.4	96.0±2.3	0.624
large-TRL TG	38.2±2.3	40.9±2.3	0.785
small-TRL TG	25.4±1.4	27.2±1.2	0.593
large-TRL CHOL	5.1±0.3	5.4±0.3	0.528
small-TRL CHOL	6.9±0.4	7.0±0.4	0.491
APOA1	66.0±1.3	64.3±1.4	0.387
Apo B	44.1±1.3	44.6±1.2	0.816
HDL	19.6±0.7	19.3±0.6	0.993
LDL	63.4±2.0	63.8±1.9	0.893
large-TRL Apo B	0.91±0.08	0.97±0.08	0.555
small-TRL Apo B	1.27±0.01	1.30±0.01	0.872

All values are mean ±S.E. Adjusted by diet, age, gender, BMI and center of origin.

### Elderly Population and Fat-loading Test

**Figure 2:**
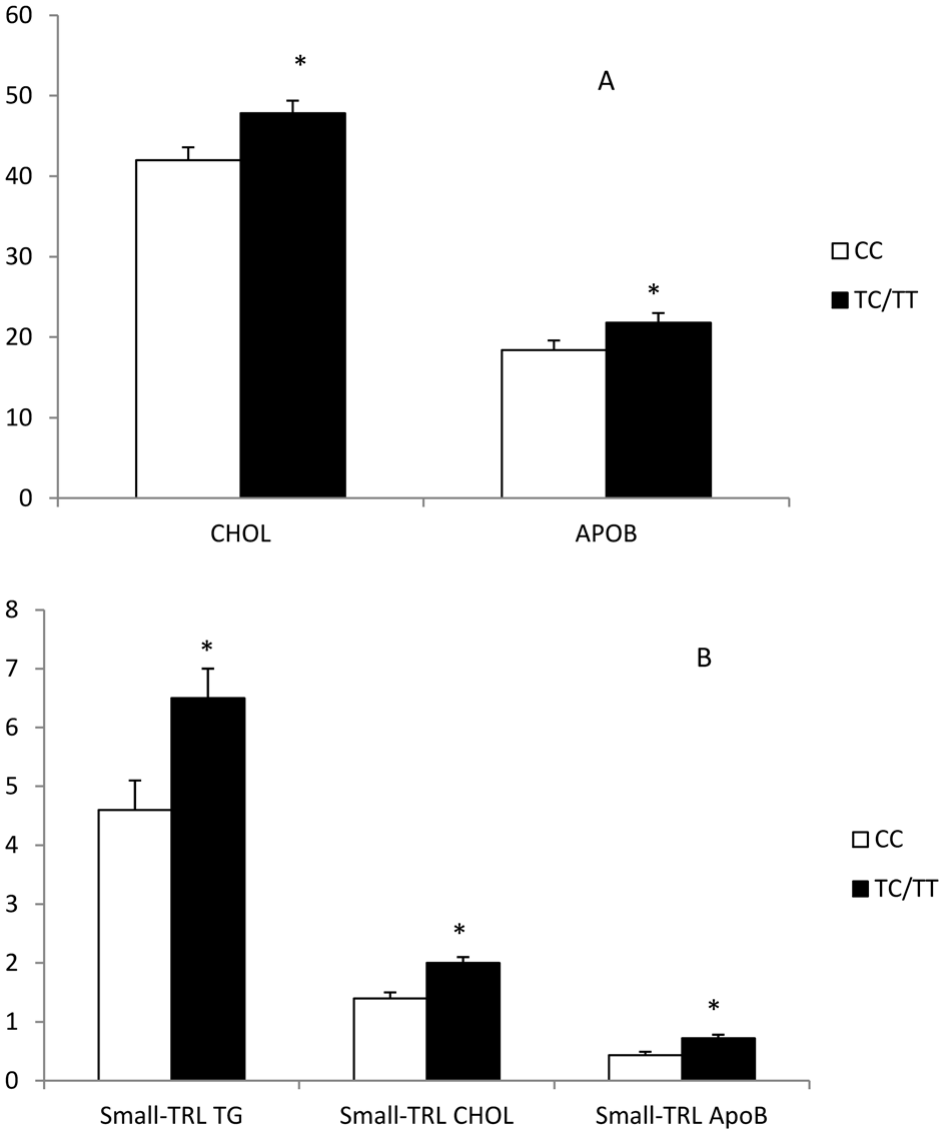
Area under curve (AUC) of Total Cholesterol and Apo B (Panel A), and Small-TRL contained Cholesterol, TG and Apo B (Panel B) depending on the rs7903146 in aged cohort (mean ±**S.E).** *p<0.05 TT vs TC/TT. Values are in (min*mg/dL)/10^3^.

**Table 4** summarizes the results of the test meal tests in the elderly persons. Carriers of the minor allele for rs7903146 had a higher AUC of total cholesterol (p = 0.028), small-TRL TG (p = 0.018); small-TRL cholesterol (p = 0.015); Apo B (p = 0.042) and small-TRL Apo B (p = 0.004) **(** **Figure 2** **)**. The repeated measures test revealed that the differences were evident at all timepoints (fasting, 1^st^, 2^nd^ and 4^th^ hours) for total cholesterol (all p<0.036), small-TRL TG (all p<0.042), small-TRL cholesterol (all p<0.044) and small-TRL Apo B (all p<0.033) and at all timepoints except 3^rd^ hour for Apo B (all p<0.042 except p = 0.070 at 3^rd^ hour). The AUC of leptin was lower, and resistin higher in the carriers of the minor allele for rs7903146. We did not find any influence of rs7903146 in HOMA-B index.

**Table 4:** AUC of the different lipid particles in the elderly persons cohort under the dominant model assumption (min*mg/dL)/10^3^.

Elderly persons cohort	Rs7903146		*P*
Short lipemia (4 hours)	CC (n = 10)	CT/TT (n = 10)	
Total TG	34.2±3.8	41.9±3.8	0.183
**CHOL**	**42.0±1.6**	**47.8±1.6**	**0.028** *
large-TRL TG	12.9±2.5	16.7±2.5	0.305
**small-TRL TG**	**4.6±0.5**	**6.5±0.5**	**0.018** *
large-TRL CHOL	1.4±0.1	1.6±0.1	0.544
**small-TRL CHOL**	**1.4±0.1**	**2.0±0.1**	**0.015** *
APOA1	35.0±1.6	36.4±1.6	0.569
**Apo B**	**18.4±1.2**	**21.8±1.2**	**0.042** *
HDL	12.2±0.7	12.1±0.7	0.940
LDL	23.3±1.5	26.6±1.5	0.150
large-TRL Apo B	0.47±0.06	0.55±0.06	0.351
**small-TRL Apo B**	**0.43±0.06**	**0.72±0.06**	**0.004** *

* p<0.05 CC vs CT/TT. All values are mean ±S.E. Adjusted by diet, age, gender and BMI.

## Discussion

Our study shows that carriers of the rare allele of *TCF7L2* rs7903146 have a disrupted lipid metabolism, which is seen in different ways depending on age and baseline conditions. Healthy young men have a lower HDL and Apo A1 response to a saturated fatty acid-rich meal, while healthy elderly persons show increased postprandial lipemia, with higher totals of plasma cholesterol and Apo B, and a higher accumulation of Apo B, cholesterol and triglycerides in small TRL lipid particles.

Variations in *TCF7L2* (particularly rs7903146) are a genetic risk factor for T2DM [13]–[15], [33]–[34]. The alterations caused by these gene variations in the glucose metabolism of diabetic persons have been largely explained by altered insulin secretion (caused by a failure in the fusion and release of the insulin-containing granules in the pancreatic beta cells) [35]. We previously showed that this beta-cell dysfunction is also present in MetS patients, affecting the dynamic markers of glucose metabolism [17]. However, the TCF7L2 pathway has also been involved in several other biological processes, like the differentiation of adipocytes and the regulation of adipokines, among others [16], [33], and the presence of the minor allele of rs7903146 gene variation has been linked to other diseases apart from diabetes, such as the incidence of cancer [36], or the severity of coronary disease [37].

Despite the fact that lipid metabolism may be a link between *TCF7L2*, adipocyte metabolism and coronary disease, few studies have evaluated fasting lipids, and the influence of gene variations on this gene and on postprandial lipids remains an unexplored field. A single previous report found that rs7903146 was linked to altered postprandial lipid metabolism only in the presence of a high PUFA intake in a sample of Caucasian origin with subjects ranging from 17 to 92 years old mostly recruited from a 3-generational pedigree database [18]. The authors identified beta-cell dysfunction as the main underlying cause, although the significance for differences in the HOMA-B index in the full cohort was nominal (p = 0.041). To further understand the significance of rs7903146 in human metabolism, we evaluated its effects in three different settings: healthy young men, middle-aged persons with MetS and healthy elderly persons, with the last two cohorts being examined after different dietary models.

The objective in choosing the first cohort (healthy young men) was to observe the possible effects of this SNP on a healthy background, where alterations in the carbohydrate metabolism were not present and the functional status of the pancreas was, in theory, optimal. In this setting, we found that the carrier status of a single minor allele for *TCF7L2* rs7903146 was not related to altered postprandial response, requiring the existence of two altered alleles (homozygote for the rare alleles) to produce altered postprandial lipids, consisting of lower HDL and Apo A1 levels during the postprandial test, which would account for the diminished donor capacity of Apo CIII, an important feature of HDL during the postprandial state.

The second cohort model (MetS) was chosen on the basis of our previous research and published data into the influence of TCF7L2 on glucose metabolism and beta-cell function [17]. In our sample, we could find no influence of these gene variations on postprandial lipemia in this population. What at first sight seemed a disappointing result may be put down to the fact that *TCF7L2* SNPs do not exert the observed effects in the presence of MetS. Nevertheless, data from a previous comparison of the three cohorts resulted in the notion that, once homogenized, these MetS patients showed altered postprandial lipemia, when compared not only to young men, but also to healthy elderly persons (unpublished data, J D-L). In this context, it is possible that the deleterious effects of rs7903146 were not evident in the MetS cohort because all the participants, even those who did not carry the “harmful” allele, may have disrupted postprandial lipemia via other physiopathological pathways.

Our final test model was a cohort of healthy elderly persons. In this cohort, the carriers of the rare allele of rs7903146 showed altered postprandial lipemia, with higher total cholesterol and Apo B, and a higher content of cholesterol, triglycerides and Apo B in small-TRL particles, accounting for a disrupted clearance of postprandial particles - which may be related to the greater severity of cardiovascular events reported for the carriers of rs7903146 [37]. We also noted higher fasting total cholesterol, total triglycerides, and high LDL concentration and Apo B molecules in this last cohort. All these features are pro-atherogenic, and may have partly determined the altered postprandial lipemia. We did not find any influence of the type of diet on the results.

When looking for potential underlying mechanisms, we studied two well-known biological properties of TCF7L2 - its influence in beta-cell function, and in the adipocyte differentiation - by measuring the main adipokines present in mature adipocytes (adiponectin, leptin and resistin). Although it was non-significant in the case of the young men, both the elderly and the young men who are carriers of the minor alleles shared an increase in resistin levels, a hormone which has been related to obesity, or an increase in insulin resistance [38]–[39]. As regards adiponectin, the T allele was related to a lower concentration in the young men’s cohort. Adiponectin participates in lipid clearance and glucose metabolism (decreasing gluconeogenesis and increasing glucose uptake) [40], and is inversely correlated to the harmful features of the postprandial state [41]. The fact that the young men with the T allele showed higher adiponectin levels, while the elderly persons did not, may partially explain the features observed in lipid clearance in the latter (and absent in the young men), which implies that young men could somehow boost their lipid clearance by increasing their adiponectin levels. Moreover, we found higher levels of leptin (an adipokine that increases in response to obesity, inhibiting appetite at brain level [42]) in the elderly persons who were homozygote for the common allele, which may be related to the higher BMI found in these persons in our sample.

Beta-cell dysfunction has been identified as the main cause by which *TCF7L2* variants may exert their effects on glucose metabolism [31], [35], and rs7903146 may impair the ability of hyperglycemia to suppress glucagon [43]. In our study, HOMA-B measures did not differ in any case between carriers and non-carriers of the minor alleles of the SNPs, in the two populations in which the SNPs influenced the postprandial lipids, which may suggest that, despite the well-established effects of rs7903146 on beta-cell function, there could be additional pathways underlying the effects of rs7903146 on postprandial lipids. At this point, a combination of the direct effects of rs7903146 on insulin secretion with those derived from the crosstalk with other regulators of the energy metabolism *via* adipokines cannot be discarded, and is something that, in our opinion, may be looked into further in *in vitro* studies.

We must be cautious therefore when extrapolating our results, because our study presents some limitations. The possibility of epistasis should be considered between the rs7903146 SNP and postprandial lipemia response in terms of possible linkage disequilibrium in the vicinity of other related genes. On the other hand, oral fat loads have still not been satisfactorily categorized, as this has mainly been studied with different oral fat loads and different timing of blood sampling. In this study, we used different methodology in order to explore the determinants and the pathophysiological aspects of exaggerated/delayed postprandial lipemia in humans. This limitation regarding the different experimental designs is a factor to be considered.

Another point to be taken into account is that, while the effects of rs7903146 in our study are circumscribed to the postprandial measurements in the young men, they are also evident in the fasting state in the aged cohort, and, hence, it may be possible that some of the effects found in this latter cohort are due to the fasting figures. Nevertheless, the postprandial lipid metabolism involves many important clearance pathways and actors, such as the lipoprotein lipase or the different apolipoproteins that could limit the extent of the fasting effects in the postprandial state. In that context, our work shows how the modifications exerted by rs7903146 in the fasting state also extend to the fed state.

To sum up, we have shown how the gene variation rs7903146 negatively influences lipid metabolism during postprandial lipemia in healthy subjects (young men and elderly persons), while we did not find any effects in MetS persons. The elderly persons also exhibited altered fasting lipids.

## Supporting Information

File S1 **Laboratory measurements (detailed description).**
(PDF)

File S2 **Comparison of the significance values based on ANOVA or Bootstrapping methods for the main findings of this article.** Bootstrapping test used the following specifications: Simple sampling method, 10,000 permutations per test and 95% of confidence interval. C.I.95%: Difference of means.
(PDF)
