# Supplementary material for: Effects of rs7903146 Variation in the Tcf7l2 Gene in the Lipid Metabolism of Three Different Populations
Source: PLoS One. 2012 Aug 20;7(8):e43390. doi: 10.1371/journal.pone.0043390 (PMC3423356; doi:10.1371/journal.pone.0043390)
Supplement: File S1 — Laboratory measurements (detailed description). (PDF) [file pone.0043390.s001.pdf]

File S1: Laboratory measurements.

Blood was collected in tubes containing EDTA to give a final concentration of 0.1% EDTA. In each center, plasma was separated from red cells by centrifugation at 1500 × g for 15 min at 4°C. The large TRL (Sf > 400) were isolated from 4 mL of plasma by a single ultracentrifugal spin (36,200 × g, 30 min, 4°C) in a type TY65 rotor (Beckman Instruments). Large TRL, contained in the top layer, were removed by aspiration and the infranatant was centrifuged at a density of 1.019 kg/L for 24 h at 183,000 × g in the same rotor. The small TRL (Sf 12–400) were removed from the top of the tube. All operations were conducted in subdued light. Large and small TRL fractions were stored at −70°C until assayed for retinyl palmitate (RP) and biochemical determinations. Analytes determined in frozen samples were analyzed centrally by laboratory investigators of the Lipid and Atherosclerosis Unit at the Reina Sofia University Hospital who were unaware of the interventions. Lipid variables were assessed with a DDPPII Hitachi modular analyzer (Roche) using specific reagents (Boehringer-Mannheim). Large and small TRL were manually extracted after centrifugation in subdued light as previously described and samples were stored at -70ºC until analyzed (1). Total cholesterol (Chol) and triglycerides (TG) in plasma and lipoprotein fractions were assayed by enzymatic procedures. APOA1 and APOB were determined by turbidimetry (2). HDL-C was measured by analyzing the supernatant obtained following precipitation of a plasma aliquot with dextran sulfate-$Mg^{2+}$, as described(3). LDL-C levels were estimated using the Friedewald formula, based on the CHOL, TG, and HDL-C values (4) for the healthy young men and the metabolic syndrome cohorts, and calculating the difference between the total cholesterol before ultracentrifugation and that at the bottom part of the tube after ultracentrifugation at a density of 1019 kg/L for the aged persons cohort .


1. **Delgado-Lista J, Perez-Jimenez F, Tanaka T, Perez-Martinez P, Jimenez-Gomez Y, Marin C, Ruano J, Parnell L, Ordovas JM, Lopez-Miranda J** 2007 An apolipoprotein A-II polymorphism (-265T/C, rs5082) regulates postprandial response to a saturated fat overload in healthy men. J Nutr 137:2024-2028
2. **Riepponen P, Marniemi J, Rautaoja T** 1987 Immunoturbidimetric determination of apolipoproteins A-1 and B in serum. Scand J Clin Lab Invest 47:739-744
3. **Warnick GR, Benderson J, Albers JJ** 1982 Dextran sulfate-Mg2+ precipitation procedure for quantitation of high-density-lipoprotein cholesterol. Clin Chem 28:1379-1388
4. **Friedewald WT, Levy RI, Fredrickson DS** 1972 Estimation of the concentration of low-density lipoprotein cholesterol in plasma, without use of the preparative ultracentrifuge. Clin Chem 18:499-502
