# Supplementary material for: Effects of rs7903146 Variation in the Tcf7l2 Gene in the Lipid Metabolism of Three Different Populations
Source: PLoS One. 2012 Aug 20;7(8):e43390. doi: 10.1371/journal.pone.0043390 (PMC3423356; doi:10.1371/journal.pone.0043390)
Supplement: File S2 — Comparison of the significance values based on ANOVA or Bootstrapping methods for the main findings of this article. Bootstrapping test used the following specifications: Simple sampling method, 10,000 permutations per test and 95% of confidence interval. C.I.95%: Difference of means. (PDF) [file pone.0043390.s002.pdf]

File S2:

Population	Variable	ANOVA with Bonferroni´s test	Bootstrapping (10,000 permutations)			
					C.I 95%	
		P value	*Comparison*	*P value*	*Lower*	*Upper*
**Aged**	Fasting Cholesterol (mg/dL)	0.013	CC vs CT/TT	0.017	-54.5	-7.5
	Fasting TG (mg/dL)	0.043	CC vs CT/TT	0.032	-54.9	2.1
	Fasting LDL (mg/dL)	0.024	CC vs CT/TT	0.028	-51.5	-6.4
	Fasting ApoB (mg/dL)	0.009	CC vs CT/TT	0.004	-36.6	-9.26
	Postprandial Cholesterol (min*mg/dL)/10^3)	0.015	CC vs CT/TT	0.024	-13.1	-1.3
	Postprandial small-TRL TG (min*mg/dL)/10^3)	0.018	CC vs CT/TT	0.020	-14.9	-3.6
	Postprandial small-TRL CHOL (min*mg/dL)/10^3)	0.015	CC vs CT/TT	0.024	-1.1	-0.16
	Postprandial ApoB (min*mg/dL)/10^3)	0.042	CC vs CT/TT	0.028	-8.7	-1.1
	Postprandial small-TRL ApoB (min*mg/dL)/10^3)	0.004	CC vs CT/TT	0.010	-0.54	-0.08
**Young Men**	Postprandial Cholesterol (min*mg/dL)/10^3)	0.053	CC vs CT	0.257	-11.0	3.0
			CC vs TT	0.125	-19.8	15.9
			CT vs TT	0.016	20.9	19.9
	Postprandial ApoA1 (min*mg/dL)/10^3)	0.011	CC vs CT	0.257	-2.5	3.6
			CC vs TT	0.125	1.2	9.1
			CT vs TT	0.016	0.8	8.6

Postprandial HDL (min*mg/dL)/10^3)	0.028	CC vs CT	0.732	-8.8	2.2
		CC vs TT	0.010	1.5	11.8
		CT vs TT	0.020	5.0	14.9
